# ChromoEnhancer: An Artificial-Intelligence-Based Tool to Enhance Neoplastic Karyograms as an Aid for Effective Analysis

**DOI:** 10.3390/cells11142244

**Published:** 2022-07-20

**Authors:** Yahya Bokhari, Areej Alhareeri, Abdulrhman Aljouie, Aziza Alkhaldi, Mamoon Rashid, Mohammed Alawad, Raghad Alhassnan, Saad Samargandy, Aliakbar Panahi, Wolfgang Heidrich, Tomasz Arodz

**Affiliations:** 1 Department of AI and Bioinformatics, King Abdullah International Medical Research Center (KAIMRC), King Saud Bin Abdulaziz University for Health Sciences (KSAU-HS), Riyadh 11426, Saudi Arabia; aljouieab@ngha.med.sa (A.A.); rashidma@ngha.med.sa (M.R.); malawad@sdaia.gov.sa (M.A.); 2 Department of Health Informatics, College of Public Health and Health Informatics, King Saud Bin Abdulaziz University for Health Sciences (KSAU-HS), Riyadh 11426, Saudi Arabia; 3 Clinical Laboratory Sciences Department, College of Applied Medical Sciences, King Saud Bin Abdulaziz University for Health Sciences (KSAU-HS), Riyadh 11426, Saudi Arabia; alhariria@ksau-hs.edu.sa; 4 King Abdullah International Medical Research Center (KAIMRC), King Saud Bin Abdulaziz University for Health Sciences (KSAU-HS), Riyadh 11426, Saudi Arabia; 5 Pathology and Laboratory Medicine, King Abdulaziz Medical City, Ministry of National Guard Health Affairs, Riyadh 11426, Saudi Arabia; khaldia@ngha.med.sa; 6 National Center for Artificial Intelligence (NCAI), Saudi Data and Artificial Intelligence Authority (SDAIA), Riyadh 12382, Saudi Arabia; rhassnan@sdaia.gov.sa; 7 Department of Community Medicine, King Abdulaziz University, Jeddah 22254, Saudi Arabia; sjsamargandy@kau.edu.sa (S.S.); tarodz@vcu.edu (T.A.); 8 Department of Computer Science, College of Engineering, Virginia Commonwealth University, Richmond, VA 23284, USA; panaali@gmail.com; 9 Visual Computing Center, King Abdullah University of Science and Technology (KAUST), Thuwal 23955, Saudi Arabia; wolfgang.heidrich@kaust.edu.sa

**Keywords:** cytogenetics, karyogram, enhancement, chromosome, CycleGAN

## Abstract

Cytogenetics laboratory tests are among the most important procedures for the diagnosis of genetic diseases, especially in the area of hematological malignancies. Manual chromosomal karyotyping methods are time consuming and labor intensive and, hence, expensive. Therefore, to alleviate the process of analysis, several attempts have been made to enhance karyograms. The current chromosomal image enhancement is based on classical image processing. This approach has its limitations, one of which is that it has a mandatory application to all chromosomes, where customized application to each chromosome is ideal. Moreover, each chromosome needs a different level of enhancement, depending on whether a given area is from the chromosome itself or it is just an artifact from staining. The analysis of poor-quality karyograms, which is a difficulty faced often in preparations from cancer samples, is time consuming and might result in missing the abnormality or difficulty in reporting the exact breakpoint within the chromosome. We developed ChromoEnhancer, a novel artificial-intelligence-based method to enhance neoplastic karyogram images. The method is based on Generative Adversarial Networks (GANs) with a data-centric approach. GANs are known for the conversion of one image domain to another. We used GANs to convert poor-quality karyograms into good-quality images. Our method of karyogram enhancement led to robust routine cytogenetic analysis and, therefore, to accurate detection of cryptic chromosomal abnormalities. To evaluate ChromoEnahancer, we randomly assigned a subset of the enhanced images and their corresponding original (unenhanced) images to two independent cytogeneticists to measure the karyogram quality and the elapsed time to complete the analysis, using four rating criteria, each scaled from 1 to 5. Furthermore, we compared the enhanced images with our method to the original ones, using quantitative measures (PSNR and SSIM metrics).

## 1. Introduction

Chromosomes, which are residents in the nuclei of all human cells (except mature red blood cells), each comprise a deoxyribonucleic acid molecule (DNA) and associated proteins. Each human somatic cell contains 23 different pairs of linear chromosomes. The study of chromosomes and their role in disease pathology is the main goal of the field of cytogenetics. Furthermore, it is considered an essential diagnostic approach for genetic disorders that are caused by chromosomal aberrations.

Cytogenomic studies include the visual observation and analysis of human chromosomes using a light microscope (also called conventional cytogenetics), determination of the presence of a specific DNA sequence using fluorescent in situ hybridization (FISH), and examination of the entire genome for copy number variation by array comparative genomic hybridization (aCGH). Although FISH and microarray studies have provided a higher-resolution analysis of chromosomal abnormalities in hematological malignancies, Giemsa-banding (G-banding) is still considered the gold standard for the conventional cytogenetic testing of bone marrow (BM) samples for most hematological malignancies, as it provides an overall visual examination of all chromosomes rather than the targeted approach provided by other techniques [1]. Furthermore, G-banding analysis reveals the presence of distinct and related clonal populations, which is an important determinant of disease prognosis [1].

Cytogeneticists are trained to manually classify G-banded metaphase chromosomes, obtained from culturing living cells, based on their length and banding pattern, which are specific to each chromosome, in order to construct the karyogram, which is the ordered appearance of chromosomes based on size and banding pattern. This is the initial step in the analytical stage of all samples received in the cytogenetics lab. As part of the development and progression of malignancies, cancer cells gain multiple numerical and structural chromosomal aberrations, including rearrangements, deletions, and duplications. The correct interpretation of these abnormalities plays a key role in the evaluation of patients with hematological malignancies for disease classification, prognosis determination, and treatment decision. However, frequently encountering metaphases of poor morphological quality and low resolution is a major complicating factor in the analytical process of BM samples. As a result, an abnormality may be interpreted incorrectly or remain unknown. Therefore, several artificial intelligence (AI) approaches to chromosome-based genetic diagnosis have been explored, many of which have focused on the development of tools that aid in the automated classification of chromosomes.

Artificial Intelligence (AI) transformed many disciplines of science, including genomics and genetics [2]. In the context of medicine and healthcare, the combination of AI with genomics and genetics, resulted in data sets that reshaped precision medicine. Machine learning (ML), a subset of AI, together with physiological genomics data, proved to be a powerful approach for precision medicine in common diseases [3]. Precision cancer care is another example of the amalgamation of AI/ML algorithms and big cancer genomics data sets to identify or discover individual genetic or genomics diagnostic and/or prognostic factors, helping to charter personalized treatment plans [4]. The most successful application of AI in medicine is medical-imaging-based clinical diagnostic tools, where computer-vision approaches utilize the imaging data to obtain sensitive diagnostics [5,6]. Genetic data of the primary sequence type (DNA or RNA) have been explored rigorously with machine learning algorithms for the task of predicting/annotating various genomic elements, such as gene-structure prediction of intron splice sites, 3′ untranslated regions, promoters, and cis-regulatory elements [7]. Besides, genetic data of primary sequence type (aligned on a reference genome) could also be transformed into images for the identification of genetic variants using deep learning methods [8]. Last but not least, the study of genetic data structural types, such as chromosomal images (cytogenetics), could also be well explored by various ML techniques to help in disease diagnosis and/or prognosis, especially in hematological cancers.

The application of AI in cytogenetics for karyotype analyses is not new. Several attempts have been made to automate, with better accuracy, the variety of tedious and time-consuming manual cytogenetic analyses, such as automatic segmentation of chromosomes [9], automatic detection of chronic myeloid leukemia (CML) using chromosome images from BM specimens of CML patients [10], automatic pairing algorithm using bone marrow cells karyograms for leukemia diagnosis [11], and automatic karyotyping of deformed chromosomes from BM cells [12]. These studies unanimously faced one major challenge in analyzing the bone marrow cell karyograms; that is, these karyogram images are of poor quality, with chromosomes appearing distorted, overlapped, and blurred. Therefore, the development of new or novel AI methods is highly needed to enhance the karyogram images to help correctly diagnose a large amount of cancer patient cytogenetic data. The enhancement has to be optimal, as the over enhancement might affect the telomeres, which are sometimes involved in abnormalities [13]. However, little progression have taken place in this context [14], so more sophisticated algorithms using different AI-based methods, especially deep neural network, are highly sought.

In this study, we used exported karyogram images from the cytogenetics system instead of using the tangled chromosomes image on the microscopic slide. We developed the ChromoEnhancer model using the CycleGAN to convert the poor-quality karyogram images into good quality. The idea of using poor- and good-quality chromosome pairs in the CycleGAN model led to a robust routine cytogenetic analysis and to accurate detection of cryptic chromosomal abnormalities. We validated our method using two different cytogeneticists, rating the enhanced karyograms with PSNR and SSIM metrics.

## 2. Materials and Methods

Karyogram images were collected from King Abdulaziz Medical City under the Institutional Review Board (IRB) number. The images were chosen to have the following features: 1—not short; 2—either of poor or excellent quality (i.e., medium-quality images were excluded).

### 2.1. Data Selection

**Figure 1:**
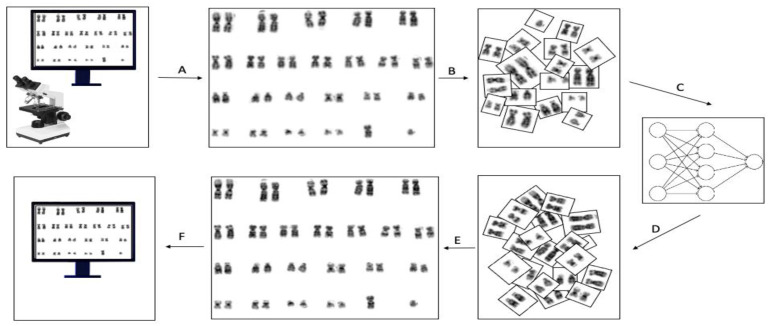
Pipeline of our method. (**A**) Karyogram taken from cytogenetics state-of-the-art system. (**B**) Cutting each pair of chromosomes. (**C**) Feeding each pair into the model. (**D**) The output of the pairs is gathered. (**E**) Reformed karyogram from the enhanced pair. (**F**) The karyogram is ready for analysis.

Following regular protocol [15], metaphase spread images were captured using the CytoVision software (Leica Biosystems Inc., Vista, CA, USA). Afterward, the karyogram was prepared and enhanced using built-in tools in the cytoVision software. We then extracted those karyograms and used them for the application of our proposed enhancement method. The CycleGAN model we designed is shape and length sensitive; thus, we aimed at maximizing the shape similarity between chromosomes. Accordingly, our problem is data-centric and requires expertise that knows both how the CycleGAN algorithm works and how to classify the quality of karyograms. We wanted to have two groups of data sets, namely, medium-size poor and medium-size excellent bone marrow karyograms. After that, we created a training data set by manually choosing 88 poor-medium karyograms and 143 excellent-medium karyograms among thousands of images to achieve the shape similarity target. A typical karyogram image consists of 23 pairs of chromosomes. We divided each karyogram image into 23 images, i.e., each karyogram generates 23 pairs, each consisting of a chromosome pair except for chromosomes X and Y, see Figure 1. Additionally, to mitigate the chromosomes’ shape and length model bias, we removed chromosomes 13, 14, 15, 16, 17, 18, 19, 20, 21, 22, X, and Y from the training set. This pre-processing step maximizes the shape and length similarity between chromosomes. However, we tested the predictive ability of the proposed model on all pairs of chromosomes. The final data set for training included 1056 poor and 1727 excellent images. Furthermore, for the testing data set, we selected another 30 poor random karyograms and divided them into 23 pairs as mentioned above. Before feeding the images into the network, we scaled each pair of chromosome images into 256 × 256 with instance norm. The final step was to enhance each of the 23 images individually and used these enhanced images to reform the karyogram.

### 2.2. ChromoEnhancer Model

**Figure 2:**
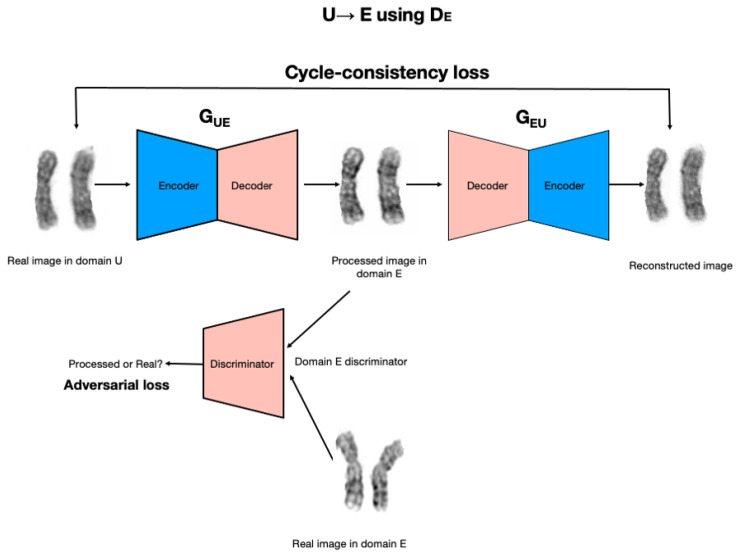
CycleGAN model architecture.

ChromoEnhancer model is a model that was designed over the CycleGAN, a deep model that we utilized in order to learn the image enhancement process. The image enhancement task is posed as an image-to-image translation problem. We used the cycle-consistent generative adversarial network approach (CycleGAN; [16]), which is an adaptation of the generative adversarial network (GAN) approach (Goodfellow et al., 2014) to image generation and translation, see Figure 2. A GAN is trained to generate an image that resembles real-world images it has in its database, without directly seeing and/or copying the images. Instead, a GAN involves two networks playing a game with each other. A generator network learns to produce images, given some random noise on the input. A discriminator network is given either a real image, or an image produced by the generator, and its goal is to learn to tell the real image from the fake one. In the process of training, the generator learns how to produce images that can deceive the discriminator.

The CycleGAN approach works with two separate databases of images (unenhanced, U, and enhanced, E, in our case) and consists of two image generators (G_UE_ and G_EU_) and two image discriminators, D_E_ and D_U_. Both generators take images on input, instead of random noise as in the standard GAN. Generator G_UE_} takes an image from database U and produces an image that is fed into D_E_ discriminator, which is tasked with learning whether the image comes from the generator, or is a real image from database E. In the course of improving both networks, the discriminator will become better at distinguishing real images from artificially generated ones. Crucially, at the same time, the generator will learn to be better at producing realistic artificial images. In particular, the G_UE_ generator will learn how to transform an unenhanced image U into an enhanced image that has a similar quality to real images from database E. In addition to the above learning process, the image generated by the G_UE_ generator is provided as input to the G_EU_ generator, and its output is compared to the original image from the U database. This ensures that the image generated by G_UE_ generator retains relevant information from the input image so that G_EU_ generator can reconstruct the input image. Similarly, a real image from database E will be provided on input to generator G_EU_, then the output produced by G_EU_ will be provided to (a) discriminator U, and (b) generator G_UE_. In the process of training the generator, G_EU_ learns to translate enhanced images into unenhanced images, and the generator G_UE_ learns to translate unenhanced images into enhanced images. Crucially, the learning process does not rely on the availability of paired unenhanced–enhanced images.

CycleGAN model uses the logistics loss for the adversarial loss and for CycleGAN Consistency Loss it uses L1 loss. We used unet_256 for network G. We set the batch size parameter to four and we applied batch normalization to each batch. Further, we set input and output networks channel to one as our chromosomal images are gray-scale images. No resize or crop was needed. For the rest of the parameters, we used the CycleGAN defaults.

### 2.3. Model Evaluation

Model evaluation was carried out to assess the quality of our enhancement in qualitative and quantitative manners. As part of the qualitative assessment, we asked two independent cytogeneticists to evaluate the images whereas quantitative evaluation focused on the use of PSNR and SSIM metrics.

#### 2.3.1. Qualitative Evaluation

Two cytogeneticists were invited to analyze 60 pairs of random karyogram images consisting of 30 normal and 30 abnormal karyograms. Each karyogram pair consists of the poor image and its enhanced version. The images were randomly ordered to create 120 karyograms. The evaluation process was blind in that the cytogeneticists were provided with the 120 karyograms without notification of their status, i.e., original or enhanced. Numerical abnormalities that can be identified without enhancement were excluded. We asked the evaluator to document the following: 1—the suspected abnormality; 2—the overall quality of each karyogram on a scale of 1 to 5 (1 being poor and 5 being excellent); 3—the sharpness of the chromosomal bands on a scale of one to five (1 being sharp and 5 being fuzzy); 4—the duration of analysis of each karyogram in seconds. We analyzed the data using a one-tailed paired *t*-test with the following hypothesis: 1—enhancing the images with our method will increase the overall quality, therefore, increasing the time required to complete the analysis; 2—enhancing the images with our method will increase band sharpness. Based on our hypotheses, we were expecting to identify abnormalities in the enhanced images that were originally missed in the non-enhanced images due to poor quality.

#### 2.3.2. Quantitative Evaluation

To quantify the quality of the enhancement, we used the two popular image quality assessment measuring metrics, peak signal-to-noise ratio (PSNR) and structural index similarity (SSIM). PSNR is defined as the ratio between the maximum possible value (power) of the ground truth signal and its corrupted noise that affects the ideal representation of the signal. SSIM on the other hand uses luminance, contrast, and structure of the images to mimic the human visual perception evaluation instead of pixel difference quantification.

#### 2.3.3. Comparing to Other Methods

To evaluate our method against some well-known methods, namely, histogram equalization (HE) and block-matching and 3D filtering (BM3D) [14], we added noise to the original image (ground truth), then we applied our method, ChromoEnhancer, along with the above-mentioned methods.

## 3. Results

**Figure 3:**
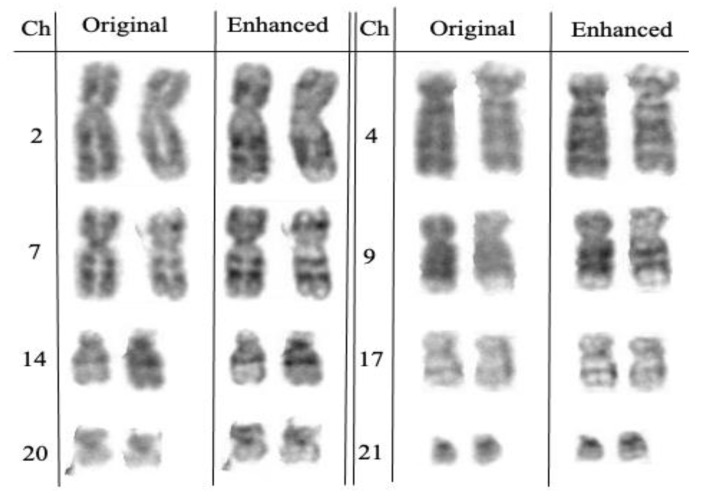
Examples of enhancing the paired chromosomes.

**Figure 4:**
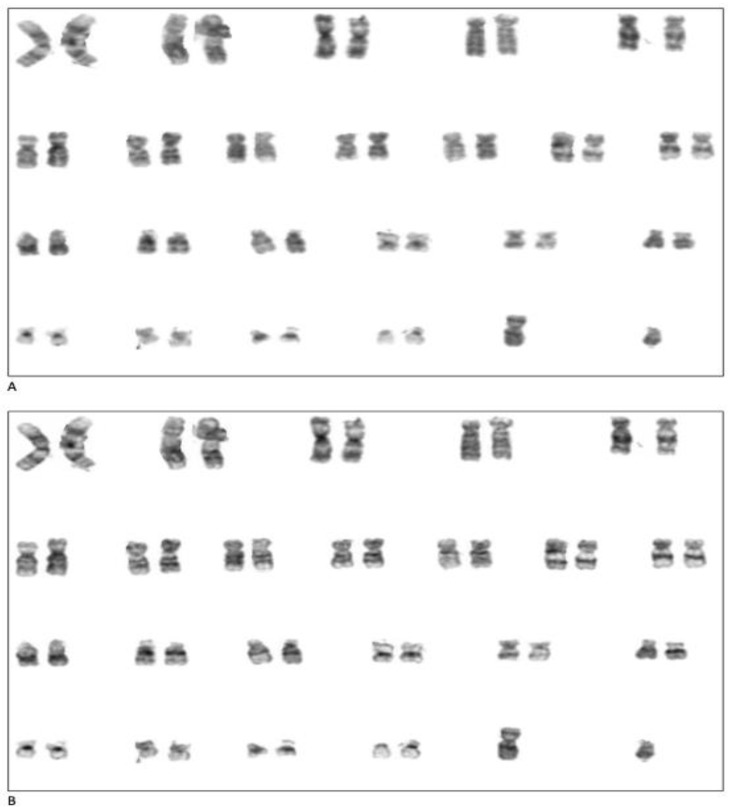
Example of whole karyogram enhancement. (**A**) Original karyogram taken from cytogenetics state of the art system, (**B**) enhanced karyogram by ChromoEnhancer.

A total of 120 karyogram images, consisting of 60 original images and their 60 enhanced images, was used to test our image enhancement model. The first stage in assessing the applicability of this enhancement model was by simple visualization of these 120 karyogram images. Overall, the original image had low contrast and low scale of image sharpness when compared to the enhanced image, which appears to have increased sharpness and better overall quality, as shown in Figure 3. In our method, we glued back the enhanced paired chromosomes to form a new karyogram, and Figure 4 shows the whole karyogram, before and after enhancement.

Further, to test our proposed hypotheses, two cytogeneticists analyzed the above-mentioned set of 120 original and enhanced karyograms, which were randomly ordered. Using a paired t-test, we compared the overall quality, the sharpness level, and the time required to complete the analysis between the original images and their enhanced version, see Table 1. The overall quality of enhanced images was significantly improved compared to their original counterparts, with a p-value equal to 0.00005 and 0.000004 for specialist one and specialist two, respectively. Moreover, chromosomal bands appeared significantly sharper in the enhanced images in comparison to the original ones (*p* = 0.02774, 0.00025). Given the overall significant improvement in the quality of the enhanced images, those karyograms required significantly more time for analytical completion than the original karyograms (*p* = 0.00184, 0.00525). The increase in the analysis time for enhanced images can be explained by the fact that good-quality chromosomes were considered for careful analysis, while poor chromosomes were not thoroughly analyzed and were often skipped due to the increase in image noise and artifacts, thus, uncertainty. It is noteworthy that the experiments were performed by two cytogenetics.

**Figure 5:**
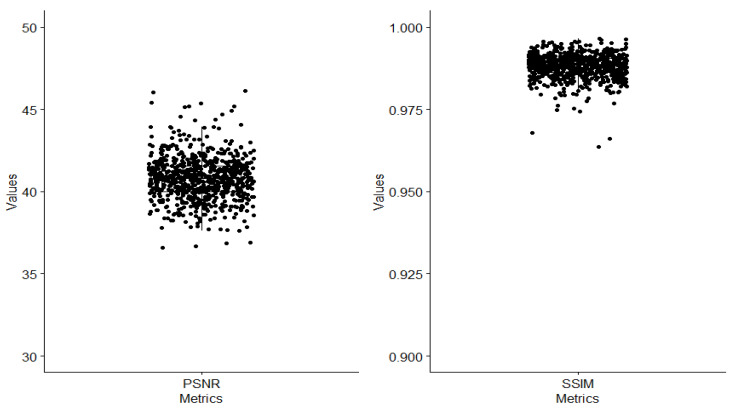
The PSNR and the SSIM of the 700 paired images.

Furthermore, PSNR and SSIM are two image quality metrics that use pixels from the paired image to check the fidelity of our paired testing image sets. To use PSNR and SSIM for quantitative evaluation, ground truth images with their distorted version are needed. We prepared 700 pairs of 24 chromosomes that have excellent quality. We used the CycleGAN model to generate poor images from excellent-quality images that we did not train the model on. Afterward, we used our model to convert the generated poor images into enhanced images. As a result, we obtained the needed enhanced images along with their ground truths. PSNR and SSIM show an outstanding improvement in the quality of the images, as shown in Figure 5. Higher PSNR and SSIM numbers indicate better quality in the enhanced image. The calculated average PSNR and SSIM were 40.795 and 0.988, respectively.

**Figure 6:**
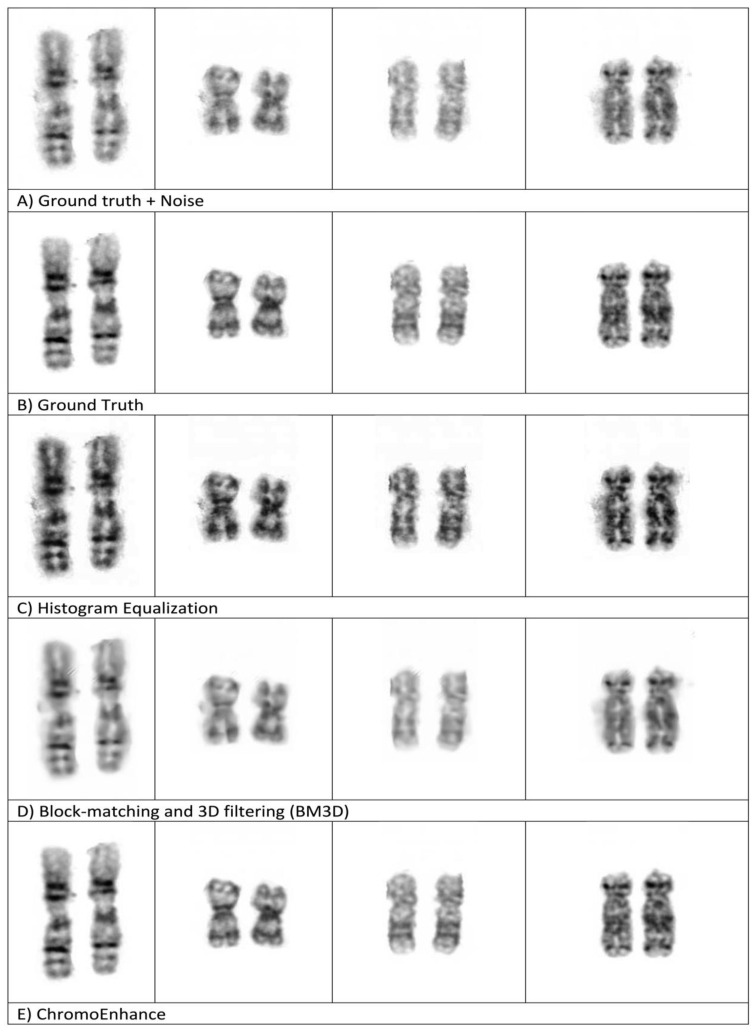
(**A**–**E**) Comparing different enhancement methods.

Finally, we visually compared the results of enhancement methods (HE and BM3D) against our method, ChromoEnhancer. Figure 6 shows four examples of paired and different length chromosomes, each being enhanced with a different enhancement method, including ours. It is obvious that our proposed method is superior and produces a better visualization effect. Figure 6 also shows that our method produces better results than the ground truth.

## 4. Discussion

In this study, we sought to improve the process of chromosomal analysis in neoplastic disorders using artificial intelligence. In this regard, we developed ChromoEnhancer, a CycleGAN-based model, to enhance the quality of karyogram images. Unlike traditional image-to-image translation methods, CycleGAN is an image-to-image translation algorithm that does not require a training set of aligned image pairs. Generally, image pairing is often difficult and labor intensive to acquire, which applies to pairing and aligning chromosomal images [16]. To the best of our knowledge, this approach is the first to enhance prepared karyograms, as opposed to common enhancement techniques on the raw captured metaphase. Additionally, our approach does not target the karyogram as a whole, but it divides it into 23 images, after which each is enhanced individually, then using the 23 enhanced images collectively to reform the karyogram. We believe this unique artificial-intelligence-based model can be used to tackle similar problems.

The significant improvement in the overall quality of the enhanced images, including the better appearance of chromosomal bands, can be of great benefit to cytogeneticists and patients, in several ways. First, the analysis of poor-quality preparations, which is a common observation in metaphase spreads obtained from bone marrow samples, is more feasible when enhanced and converted into a better-quality karyogram. This, in turn, helps in decreasing the number of reported sub-optimal results due to the inability to complete the analysis of poor-quality images. A sub-optimal result is frustrating, as it puts the patient through an invasive procedure of obtaining another bone marrow sample. Second, analyzing a karyogram that is of excellent quality helps in the correct identification of the underlying abnormality, thus, providing better management of the patient’s condition. Third, analyzing chromosomes with sharp bands aids in the accurate identification of breakpoints, which is often a challenging task in poor-quality preparations. Although the application of our model increased the time required for the analysis due to the enhanced quality, this might decrease the need for an additional sample to complete the analysis.

We believe that our model is robust; however, as with any AI model, it may be subjected to unintended bias [17,18]. CycleGAN models are good at improving the visual quality of images, but like GANs, in general, they have no guarantees that the details will match a ground truth exactly. Therefore, ChromNet must be used as an assistant tool to improve the efficiency of the cytogenetics laboratories. Additionally, to boost the applicability of our model in clinical practice, it needs external cross-study validation [19].

**Figure 7:**
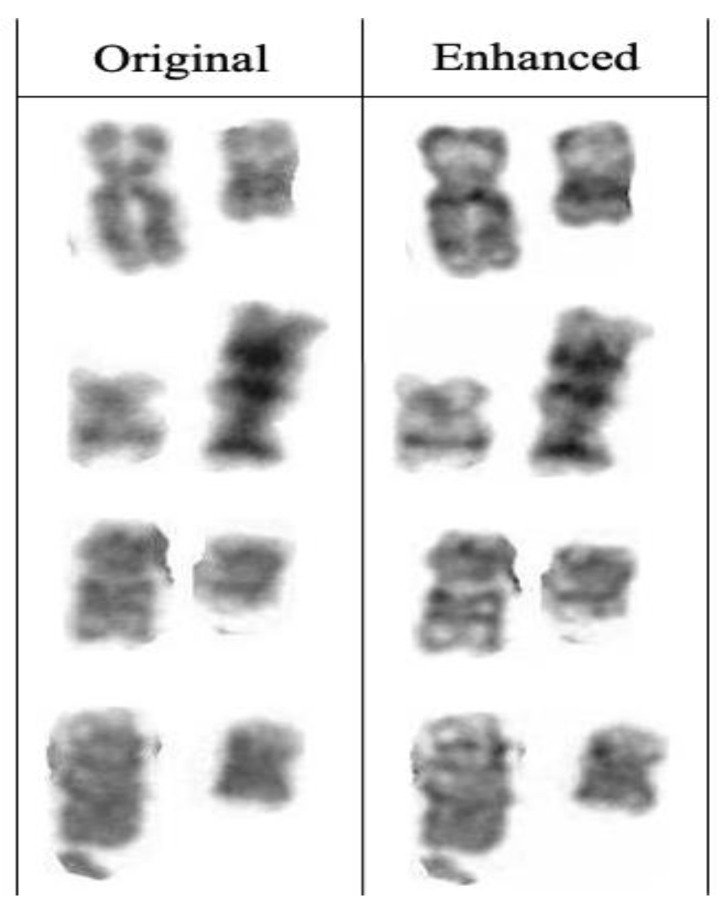
ChromoEnhancer Enhances the paired chromosomes with big abnormalities without completing or removing the missing or extra parts, respectively.

We built our model to be robust by not adding or deleting information to the karyogram. Such information could be completing a missing part of abnormal chromosomes (Figure 7) or auto-complete the short chromosomes to match long chromosomes. 

## 5. Patents

This manuscript is part of our patent application in the US patent office: App. 17/204,286 [20].

## Figures and Tables

**Table 1:** This table shows a significant p-value on the following hypothesis: 1. Enhancing the images with our method will increase overall quality; 2. Enhancing the images with our method will increase the sharpness; 3. Enhancing the images with our method will increase the analysis time.

Cytogeneticist	Hypothesis 1	Hypothesis 2	Hypothesis 3
Specialist 1	0.000004	0.02774	0.00184
Specialist 2	0.00005	0.00025	0.00525

## Data Availability

The data sets generated during and/or analyzed during the current study are available from the corresponding author on reasonable request. The prediction model used to develop ChromoEnhancer is publicly accessible through this GitHub repository https://github.com/bokhariy/ChromoEnhancer (accessed on 3 April 2022).
